# Health service utilization, perspectives, and health-seeking behavior for maternal and child health services in the Amazon of Peru, a mixed-methods study

**DOI:** 10.1186/s12939-019-1056-5

**Published:** 2019-10-15

**Authors:** Christopher M. Westgard, Ally Rogers, Giselle Bello, Natalia Rivadeneyra

**Affiliations:** 1 Department of Research, Elementos, Lima, Peru; 2 Department of International Health, Bloomberg School of Public Health, Johns Hopkins University, Baltimore, MD USA

**Keywords:** Health services, Health service utilization, Health-seeking behavior, Maternal health, Child health, Implementation research, Amazon, Peru, Health systems, Micronutrient supplements, Family planning

## Abstract

**Background:**

Various factors influence health service utilization at the community level. Research on the barriers to uptake of local health services is essential to reduce maternal and child mortality and morbidity. The Amazon region of Peru has some of the poorest health indicators in the country. The current study set out to better understand the health-seeking behavior and perspectives of mothers in Amazonian communities, exploring individual- and contextual-level barriers for seeking care at local health facilities for common maternal and child health issues.

**Methods:**

The study employed a mixed-methods design by conducting 50 structured interviews with mothers of children under the age of 4. The study took place in 5 communities in Loreto, Peru. The quantitative data was analyzed with descriptive statistics to identify participants’ socio-demographic characteristics and reported utilization of health services. The qualitative data was analyzed in three rounds: inductive codebook development, application of the codebook, and thematic synthesis to contextualize the quantitative results and better understand the perspectives of the mothers regarding maternal and child health issues and the local health services.

**Results:**

Overall, reported health service utilization among study participants was relatively high. However, the mothers identified several individual- and contextual-level factors that may affect their experiences and the health-seeking behaviors of other mothers in their communities: (i) embarrassment, fear, and trust, (ii) insufficient number and poor attitudes of health personnel, (iii) limited supply of basic medicines and materials in the health facility, and (iv) low demand for family planning services and limited awareness of adolescent-specific services.

**Conclusion:**

Several findings in the current study reflect the reduced conditions of health services, while others display that many mothers maintain a positive outlook on the health services available to them and are proactive in the care of their child. The study provides valuable insight into the use of local health services and the common perspectives that are hindering further uptake at the community level in the Amazon of Peru, with important implications for health policy.

## Background

Maternal and child morbidity and mortality from preventable diseases can be dramatically reduced with inexpensive, integrated care at the community level. Availability and use of local health services are instrumental in reducing child deaths in low- and middle-income countries [1]. Effective care can only occur when there is both sufficient capacity of local health services and appropriate health-seeking behavior by caregivers (e.g. parents and guardians). Health-seeking behavior (HSB) has been defined as “any action or inaction undertaken by individuals who perceive themselves to have a health problem or to be ill for the purpose of finding an appropriate remedy” [2]. The Integrated Management of Childhood Illness strategy by the World Health Organization (WHO) highlights health-seeking behavior by caregivers as a key activity to improve family and community health [3]. Research on barriers to access and use of the formal health system has been identified as the highest research priority to encourage appropriate health-seeking behaviors among caregivers and reduce child mortality from childhood illnesses by the Child Health and Nutrition Research Initiative (CHNRI) and the Global Action Plan for the Control of Pneumonia and Diarrhea of the United Nations Children’s Fund (UNICEF) [4, 5].

The current study will draw on concepts from the Behavioral Model of Health Services Use, which has been extensively used to describe the complex series of steps that individuals take towards health care, to highlight the individual- and contextual-level factors that can act as barriers in the complex process for seeking care at health facilities [6, 7]. Various factors influence health-seeking behavior, such as an individual’s socio-demographic characteristics, their awareness of existing services, and their perceptions of those services, as well as cultural norms, disease patterns, and characteristics of the local health services system, including its personnel [8]. Previous studies have demonstrated a well-established link between socioeconomic status and health, including health prevention, household sanitation, diet, and utilization of health services [9–20]. Maternal education is also a reoccurring significant social determinant associated with maternal and child health and health care use [21–23]. Additionally, a lack of sufficient recognition of local customs and indigenous medical practices by the health service providers can influence the use of government-provided health services [24, 25]. With regards to the local health care system, the access costs of health services (monetary and time) can be a significant burden for impoverished populations and a barrier to their utilization. Furthermore, attitudes and behaviors of health care providers in these facilities can influence health-seeking behavior by the local population. Behaviors such as verbal abuse, neglect, absenteeism, lack of privacy, and authoritarian attitudes can deter patients from using health services [26]. It is important to explore which of these barriers are the most influential to the uptake of health services within local contexts. Exploration of caregiver’s perceptions of the formal health system can provide insight into which barriers create the greatest burden and should be addressed.

The department of Loreto, in the Amazon region of Peru, has some of the poorest maternal and child health indicators in Peru. In 2017, the infant mortality rate in the department was reported to be 30 deaths per 1000 live births, the highest in the country [27]. In 2017, the prevalence of child anemia was reported to be at 49.9%, and child chronic malnutrition prevalence was23.6% [27]. Loreto also has considerable annual incidence of diarrheal disease in children (18.8 cases per 1000 inhabitants) and pneumonia in children (191.6 per 10,000 inhabitants) [28, 29]. Child developmental delay in the region was reported to be 26.7% [30]. Finally, the use of maternal health and family planning services in the region is limited. Only 83.5% of women received prenatal care and 71.5% of women gave birth in a health establishment [31]. Furthermore, only 63% of women in Loreto report using any method of contraception, with 43% using modern methods of contraception to prevent pregnancy [27]. Loreto also has one of the highest rates of adolescent pregnancy in Peru, with 30.4% of adolescents reporting they have had a child or were at one time pregnant [27].

The population of Peru below the poverty line has access to the Seguro Integral de Salud (SIS), a free health insurance that provides essential health care through public health facilities, including diagnostic services, essential generic drugs, integrated maternal and child health services, and emergency care services. For the population of the current study, the local health facilities offer health care and pharmaceuticals to all people free of charge through SIS, regardless of socioeconomic status, because the region has poverty levels higher than 60% [19]. Additionally, a national program known as Programa Juntos provides conditional cash transfers to most families in the region. To receive the funds the mothers must bring their child to their grow monitoring check-ups, and older children must attend school [32]. Nonetheless, even when services are free of charge, issues of transportation, stock of materials and essential medicines, and attendance of health professionals can create barriers to access of care [33].

The authors of the current study found no research that explores the perceptions of caregivers regarding local health services in the context of the Peruvian Amazon. For health care services to be effective, policy makers need to understand the drivers and barriers of health-seeking behavior within a population, especially in locations with diverse cultures and geographical contexts such as the Peruvian Amazon [34].

The current study set-out to address the research gap by exploring caregivers’ perceptions to better understand the individual- and contextual-level factors that influence the health-seeking behavior of caregivers in Amazonian communities of Peru. The research identifies barriers to the uptake of formal health services for care of maternal and child health. The study focused on the health-seeking behavior of mothers of young children (< 4 years old) when facing issues of maternal and child health and common illnesses that occur in their communities and investigated the use and experiences of mothers engaging with a variety of health services.

## Methods

The study included formative research of mixed-methods interviews to better understand the utilization and perspectives of local health services for maternal and child health services by mothers.

### Study area

**Fig. 1:**
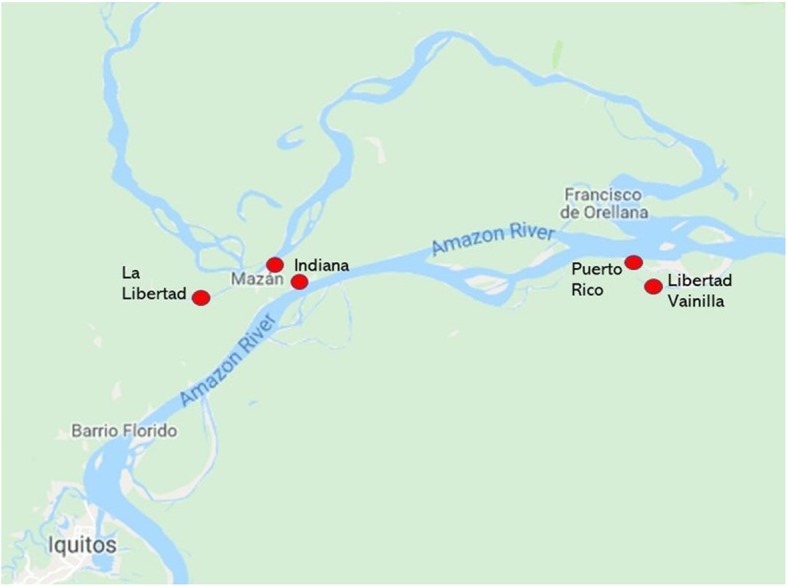
Map of Location of Communities [37]

The study took place in the districts of Mazan and Indiana in the department of Loreto, Peru. The district of Mazan has 76 communities in its jurisdiction and the district of Indiana has 57 communities. The communities in both districts are only accessible by boat from the Amazon River and are ethnically mestizo. The dominate language spoken is Spanish, however, some leaders and members of the community also speak Kichwa. The people are largely farmers and fishermen, as most families have a small plot of land where they produce mostly bananas and cassava. Mothers predominantly stay home to care for their children or sell produce and handicrafts from in-home stores or at local markets. The distance of the communities from Iquitos, the department capital, varies greatly, ranging from 1 to 8 h [35]. The communities were chosen to participate in the current study using purposeful sampling based on geographic location, health facility type, and community size to represent the diversity of the districts’ communities. The locations of each community, distance from Iquitos, population size, and sample size for the study are included in Table 1**.** The location of the communities and relative distance to the department capital, Iquitos can be seen below in Fig. 1.

**Table 1:** Communities and sample size included in the study

District	Community	Health Facility	Travel time by boat from Iquitos	Population ^a^	Sample Size
Indiana	Indiana	Level I-3	2 h	6000	15
	Libertad Vainilla	Level I-1	8 h	90	8
	Puerto Rico	None	8 h	60	4
Mazan	Mazan	Level I-3	2 h	3725	18
	La Libertad	Level I-1	3 h	240	6
	Total				50

^a Sistema de Informacion Geografica [Internet]. Instituto Nacional de Estadistica E Informatica; 2017^ [36]

The communities in the study had either a Category I-3 health center, a Category I-1 health post, or no health facility located in the community (as shown in Table 1). Defined by the Peruvian Ministry of Health, a Category I-3 health center is staffed with a family doctor, nurse, and obstetrician. A Category I-1 health post is staffed with a nurse technician [38]. The health facilities in the region offer a package of standard services at no cost to all individuals with their national identification card. The services include: prenatal checkups, iron-folic acid tablets for pregnant women, micronutrient powder for children under 4, child growth monitoring checkups, labor and delivery, vaccines, family planning, attention and medication for acute illnesses (ex. diarrhea, fever, cough), and a comprehensive set of adolescent health services with the aim to reduce adolescent pregnancy, which includes general health checks and family planning consultations.

The health facilities in each community in the region have registered Community Health Agents (a volunteer community member with basic training in health promotion) to help with campaigns. Additionally, some of the municipalities in the region have created and support their own Community Health Agent program. At the time of the study, the Community Health Agent program from the health facility focused on campaigns to detect and refer cases of malaria. The Community Health Agent program from the municipalities conducts home visits with mothers of children under 3 year to focus on health promotion. All the communities of the current study have both Community Health Agent programs in place. The extent to which the Community Health Agent program is operational varies greatly by community. There are no health-related programs from non-governmental organizations in the study communities.

### Study population and sample

The study population included mothers of children below the age of 4 living in the five study sites within the department of Loreto, Peru. Respondents for the 50 structured interviews were recruited using purposive sampling. In order to identify participants, the research team first met with community health agents to draw a map of their community and identify households with young children. The researchers then randomly split up and were assigned to different sections of the community to conduct the interviews. The investigators knocked on the doors of houses within their assigned sections and asked if the family had a child under the age of 4, and if their mother was home. If the household met this inclusion criteria, the researchers explained the study to the mother and presented the informed consent form. If the mother agreed to participate, the interview was conducted. The sample size for each community was determined based on saturation; the investigators ceased recruitment when the information reported by participants became redundant and no new themes were emerging.

#### Quantitative

The quantitative questionnaire included 42 closed-ended questions regarding demographics, social determinants related to health, and health service utilization regarding prenatal care, child birth location, child growth monitoring checkups, health facility accessibility, preferred source of health information, and family planning awareness and availability. The quantitative survey lasted between 15 and 20 min.

#### Qualitative

The qualitative questionnaire included 36 open-ended questions. The questions contextualized participant responses to the quantitative questions, providing the mother the opportunity to explain her health-seeking decisions and express her opinion regarding the health services and health-seeking behavior of other mothers in her community. In order to address potential social desirability bias of the mothers’ responses, participants were also asked why some people in their community may not utilize specific services. Sample questions include: *“If your child is very sick with a cough or fever, what do you do?”* and *“Why do you think some people do not take their children to CRED (child growth monitoring check-ups)?”.*The qualitative survey lasted between 20 and 30 min.

### Instruments and data collection

The mixed-methods questionnaire was administered using the Magpi data collection application [39]. This structured interview guide included 78 questions, 42 quantitative and 36 qualitative questions. The questions were directed to the mother and referred to her health service utilization and decisions during her most recent pregnancy and for the care of her youngest child. If the youngest child was too young for some of the questions, such as use of micronutrient supplements, the researcher asked about the care of the next youngest child. The interview guide and questions were validated with community members from the study communities before formal data collection began.

Data collection took place in January and February of 2018. The interviews were conducted by the authors of the current study, all of whom speak Spanish and have received extensive training on conducting interviews. Interviews were conducted in Spanish in participants’ homes. Each interview lasted approximately 45 min. Written informed consent was obtained prior to beginning the interviews. Participant responses were recorded by the interviewer in Spanish.

The quantitative questions were presented first followed by the qualitative questions. For the qualitative questions, the interviewers were directed to ask follow-up questions to identify why the mother responded in that way and what other opinions she may have.

### Data analysis

The survey data was exported from Magpi and then organized, cleaned, and analyzed by hand using Microsoft Excel [39, 40]. The quantitative data was analyzed to obtain descriptive statistics, including frequencies, means, and ranges of responses [41].

Analysis of the qualitative data was structured in three rounds. First, a codebook was developed with topical codes based on the interview questions and inductively created response codes based on the first 10 participants’ responses. In the second round, the first 10 transcripts were double coded to ensure coder agreement, discussing any discrepancies in the codes and expanding or refining codebook definitions as needed. Lastly, the codebook was applied to the remaining transcripts, and the research team met to review the quotes for each code, synthesize the information, and compare within and across transcripts to select key quotes that best reflected each theme. Translation of selected quotes were reviewed by a second translator to ensure accuracy.

The quantitative and qualitative results are both presented to triangulate and confirm the responses provided. By triangulating the responses, we can see the mothers’ experiences and opinions regarding a health service along with the portion of respondents that report utilization of that health service.

## Results

The results illustrate the participants’ health-seeking behavior and perspectives on issues of access and availability of health services and health information, prenatal care and childbirth, child growth monitoring checkups and use of micronutrient supplements, childhood illnesses, and family planning and adolescent health services.

### Quantitative results

The quantitative results are divided into four sections; demographic information, preferences of participants, health facility characteristics, and health services utilization.

#### Demographics

A total of 50 respondents were included in the study. All participants that were invited to participate in the study agreed to receive the survey. The average age of the respondents was 26.1 and the average number of children for the respondents was 2.9 [1–10]. Each mother had a child under the age of 4 years. The demographic information is summarized in Table 2.

**Table 2:** Demographic information of study participants

	*N* = 50	
	Range	Mean/Percentage
Age of mother	16–47	26.1
Number of children of the mother	1–10	2.9
Age of youngest child (in months)	1–36	15.3
Education of Mother:	–	–
Only Primary School	–	22%
Attended secondary school but did not finish	–	51%
Finished secondary school	–	27%

#### Preferences and awareness

The participants indicated their preferred source of health information and advice; 31% preferred to speak with a community health agent, 60% preferred to speak with a medical professional in the health facility, and 9% preferred both equally. The results also display the participants’ awareness of various modern methods of contraception, the availability of those methods for free at the health facility, and the existence of the health center’s adolescent health program. Most participants (90%) were aware that contraceptives are provided for free at the health facility, but only 26% were aware of the health center’s adolescent-specific service package. The responses are summarized in Table 3.

**Table 3:** Preferences of Participants and Awareness of Health Services

	*N* = 50
	Percentage
Preferred Information Source:	
Prefers speaking to a community health agent	31%
Prefers speaking to a medical professional in the health facility	60%
Prefers speaking to both equally	9%
Awareness of the following contraceptives:	
Birth control pills	78%
Contraceptive injection	88%
Contraceptive implant	50%
Condom	42%
Emergency contraceptive pill	2%
None	6%
Other	
Aware of free contraceptives offered in health facility	90%
Has heard of adolescent-specific services	26%

#### Health facility characteristics

The participants were asked about specific characteristics of the health facilities related to operating hours and contraceptives offered. Here, 41% of participants responded that the health facility is sometimes closed when they visit. The most common contraceptives offered to mothers at the health facilities were the contraception injection (80%) and birth control pills (42%). Additionally, 12% of mothers reported that the health facility does not offer any contraceptive services. The responses are summarized in Table 4**.**

**Table 4:** Reported characteristics of health facilities

	*N* = 50
	Percentage
Health Facility Accessibility	
Health facility is always open when visited	59%
Health facility is sometimes closed when visited	41%
Contraceptives offered at the health facility:	
Birth control pills	42%
Contraceptive injection	80%
Contraceptive implant	30%
Condom	10%
Emergency contraceptive pill	0%
None	12%

#### Health services utilization

The quantitative survey also provided information about the mothers’ use of the local health services for different issues pertaining to maternal and child health. Overall, reported health service utilization among study participants was relatively high, with 82% reporting attendance to at least 8 prenatal checkups and 96% reporting attendance to all scheduled child growth monitoring checkups. The reported utilization rates are summarized in Table 5.

**Table 5:** Reported use of health services

	*N* = 50
	Percentage
Went to at least 8 prenatal checkups	82%
Took prenatal vitamins during pregnancy	98%
Goes to all scheduled child growth monitoring checkups	96%
Gives/Gave child micronutrient powder	93%
Child Birth Location	
Gave birth in Regional Hospital	32%
Gave birth in Level I-3 health center	49%
Gave birth in Level I-1 rural health post	11%
Gave birth at home	8%

### Qualitative results

The qualitative portion of the survey aimed to contextualize the quantitative responses summarized above. It included questions on satisfaction with the services provided and delved into the mothers’ perspectives on various maternal and child health services. Across the health topics and services mentioned, key themes emerged from the data as the main individual- and contextual-level influences on the health-seeking behaviors of mothers in these communities: (i) embarrassment, fear, and trust, (ii) insufficient number and poor attitudes of health personnel, (iii) limited supply of basic medicines and materials in the health facility, and (iv) low demand for family planning services and limited awareness of adolescent-specific services. These themes did not appear to differ between the study communities. These themes are presented in-depth below.

#### Embarrassment, fear, and trust

Many respondents reported feelings of embarrassments, fear, and trust as major influences on the health-seeking behaviors of mothers in their community. While 82% of mothers indicated that they went to at least 8 prenatal checkups at the health facility (Table 5), the majority mentioned that some women in their community do not attend the prenatal checkups because of embarrassment. They mentioned that adolescent mothers in particular might feel the most embarrassment in going to the health clinic.

-
  “*Sometimes because they are afraid to go to the hospital and it’s safer at home, more like [they feel] embarrassment, because they aren’t used to having others look at them … ”*– Mother, Mazan
-
  *“I like to see if [my baby is] growing well, there are some husbands that don’t want because the health technician is a man and they want a woman, some are ashamed, the adolescent mothers feel ashamed.”* – Mother, Libertad Vainilla

The respondents were also asked about their use and opinions of the prenatal vitamins that they receive at these check-ups. The majority of respondents indicated that the prenatal supplements were always accessible when they wanted them, with 98% reported use during their latest pregnancy (Table 5). When asked the reasons why some community members do not take the supplements, the most common reasons cited were fear that the baby would develop excessively and make the delivery painful and complicated.

-
  *“The majority say [they don’t take their supplements] so that their baby [doesn’t] grow big and it can be born normally instead of [needing a] cesarean section.”* – Mother, Indiana

Interestingly, due to this fear, several respondents stated that they prefer to drink natural or traditional supplements, such as masato (a fermented cassava drink). This reveals important cultural practices that influence women’s decision to actually take the prenatal vitamins they receive them from the health facility, and may subsequently impact the health of the mother and her baby.

-
  *“Some receive [prenatal vitamins] and don’t take them, [they say] ‘I forgot or it gives me diarrhea,’ they drink masato and don’t take the vitamins, they think masato is enough vitamins.”* – Mother, Libertad Vainilla

After birth, caregivers in Peru are directed to take their newborns and children for free regular growth and development monitoring checkups according to an established schedule, known as the “Controles de Crecimiento y Desarrollo,” or CRED. Almost all of the mothers (98%) reported attending all of these checkups (Table 5). When asked why some caregivers do not go to CRED, they mentioned that they don’t want to give their children vaccines for fear of them making their child sick. Interestingly, some respondents also placed blame on the caregivers, stating that they are irresponsible or too busy to go.

-
  *“They are irresponsible parents who do not care about their babies’ health. When [the health technicians] test their [child’s] hemoglobin and say they are anemic, [the parents] say it’s a lie.” –* Mother, Mazan
-
  *“Sometimes moms don't take them, and why don't they take them if they live close? If I ask them they say they've forgotten, or don't have time, they're at the farm or they're washing [clothes].” –* Mother, Libertad Vainilla

Lastly, the concept of trust surfaced during discussions related to the mothers’ preferred source of health information in their community. The 61% of respondents who reported a preference for receiving information from a health professional at the health facility (Table 3) mentioned that they had more trust in health professionals due to their educational background.

-
  *“Better to go to the health post. In the post they know more, they went to school for this.”* – Mother, Indiana

On the other hand, those who preferred speaking with community health agents (39%) cited having greater interpersonal trust with these workers because they are members of their community. This reveals important factors of interest to community members that may affect their acceptance of health information and advice from certain personnel.

-
  *“I prefer a promoter. Sometimes at the health post I feel embarrassed, but with my neighbors I have more trust.*” – Mother, Indiana

#### Insufficient number and poor attitudes of health personnel

Because the respondents reported high use of the health services available, the researchers were able to explore their experiences at the facilities more in-depth, as well as the facilitators and barriers they encountered when seeking care. One repeated theme was insufficient staffing, reflected in childbirth and other health services. Regarding childbirth, 92% respondents chose to give birth in a health facility and explained that they felt it was the safer option, especially when their pregnancy had a complication. However, of the few respondents that gave birth at home, half indicated that this decision was because the health care worker was not in the health facility at the time of going into labor. Other respondents also mentioned staffing issues in relation to the CRED services, citing that the inadequate number of health personnel resulted in limited operating hours and long wait times. As one mother explained:

-
  *“[More availability] would be good, but he is the only one at times and many people need him. For example, a young woman who’s very sick, a woman who needs to give birth, etc. may need to wait.” –* Mother, Puerto Rico

In addition to these challenges, some mothers mentioned experiencing time related barriers (long wait times, rushed appointments) and impolite staff in the health facilities.

-
  *“Sometimes they don’t treat you well, they don't have patience. If they call you by name and you don’t go right away they get angry.”* – Mother, Mazan
-
  *“I get treated too fast or with two or three children at the same time.”* – Mother, Indiana

The limited number and poor attitudes of health personnel is of concern as it affects the accessibility of health services in these communities, the quality of care received by women and their children, and the community’s perception of the health services provided.

#### Limited supply of basic medicines and materials in the health facility

The respondents were also asked about the availability of needed materials and medications at the health facility. The majority of respondents reported that the health facilities often lack general supplies to treat common illnesses (e.g. antibiotics or fever/cold medicine).

-
  *“Sometimes there is no medicine, or shots and pills and many things necessary for fever and flu, for everything. They also don’t have vaccines sometimes.”* – Mother, Libertad Vainilla

Some respondents mentioned that since the health facilities lack these basic medicines, they must buy them at their local pharmacies or they must travel even farther, to Iquitos (the department capital), to buy the necessary medicines. As one respondent mentioned, this can pose a significant economic barrier to receiving the care they need.

-
  *“Sometimes I don’t have the money to buy what I need and the health center doesn’t have it.”* – Mother, Mazan

#### Low demand for family planning and limited awareness of adolescent-specific services

As shown in Tables 4, 90% of respondents were aware that they could obtain family planning counseling and contraceptives for free at the health facility. Additionally, some respondents indicated that they had previously utilized these services and received family planning counseling. Of those who received this counseling, many felt that the options had been explained well to them.

Respondents were then asked if they had ever wanted to access contraception but had not been able to, and the reasons why. Just over half of the respondents indicated that they had never had trouble accessing contraception, as contraceptives were always available at the health facility. On the other hand, many responded that they had simply never sought to access contraception, signifying a low demand for family planning services rather than a lack of awareness or supply of contraceptive methods.

-
  *“I always could [access]. I never took care of myself to prevent a pregnancy, [this is] the first time.”* – Mother, Mazan

Respondents were also asked if they were aware of the health services available specifically for adolescents at their local health facility, and what these included. As presented in Tables 3, 26% of respondents were not aware of these services. Some respondents mentioned services available for family planning and distribution of contraceptives. Looking at adolescent mothers specifically (the target demographic), the pattern was the same; few had heard of any services available, and only a quarter of adolescent respondents mentioned family planning services available to them. Only one adolescent mother had utilized the services and explained in detail what they consisted of.

-
  *“Yes, they told me to prevent [pregnancy] with methods which are many, they give talks, they give workshops. Every 3 months, to young people they give talks in the high school, or house to house, or in a neighborhood.”* – Adolescent mother, Indiana

## Discussion

The study population in the department of Loreto, in the Amazon region of Peru showed great perseverance and drive to achieve improved maternal and child health for themselves and their families. The population in the region faces many barriers that make them vulnerable to disease and mortality, such as poor levels of health education, barriers to health care, and poverty [42]. Despite these barriers, reported service utilization was relatively high among the study population. The current mixed-methods study shows the interaction between the perseverance of the mothers and the shortcomings of the local health services. The majority of the mothers reported that they were content with the health services they receive in their community. However, 41% indicated that the health facility is sometimes closed when they arrive, 58% indicated that the health facility often lacks essential material and medicines, and 31% preferred to speak to a community health agent rather than their local health professional. Additionally, most reported that they attend their child growth monitoring checkups (93%), but many indicated that long wait times and insufficient operating hours at the health facilities made it difficult for some in their community to access these services. This displays perseverance by the study mothers to go to health checkups even in unfavorable conditions. Below, some of the key themes with regards to individual- and contextual-level barriers to care that emerged from the findings are explored, namely: (i) embarrassment, fear, or trust (ii) insufficient number and poor attitudes of health personnel (iii) limited supply of basic medicines and materials in the health facility, and (iv) low demand for family planning services and limited awareness of adolescent-specific services. Additionally, other aspects for further discussion and consideration are presented.

Across several health services, the respondents’ indicated that some mothers in the community avoided the health facilities because they felt embarrassment (for example, during the prenatal health checks). Others expressed mistrust or feared an adverse effect from one of the services offered (for example, that her unborn child would grow too large if she took prenatal vitamins).

As a counterpart option to speaking with health personnel, community health workers have been shown to be effective in the region at delivering health messages and forming positive relationships with caregivers in these communities [43]. Community health agents have also been proven effective at improving levels of care-seeking for childhood illnesses [44–46]. In another study, 64% of the caregivers surveyed had talked to a community health agent in the last month [30]. However, in the current study, there was some doubt expressed as to the effectiveness of community health agents’ knowledge and dedication. For example, as indicated by one mother in Indiana, “*… sometimes the promoter doesn’t know, the promotor who comes hardly comes.*” This represents an opportunity to improve the community health agent program in these communities.

The insufficient number of health personnel, poor attitude of some health staff, insufficient operating hours, long wait times, and lack of basic supplies and medicines in the health facilities reported by respondents all illustrate important access barriers for mothers’ in these communities, which can hopefully be addressed through corresponding policy changes. Indeed, a nationwide survey revealed that hospitals and health centers are the public spaces where people most feel discriminated [47]. This suggests that capacity building for health personnel could improve patient interactions with health services. Regarding the lack of supplies and medicine that sometimes occurs in the communities, this may speak to the logistical complications of consistently reaching these communities. The respondents mentioned that sometimes even their local pharmacies lack necessary medicines, and they must travel to the department capital of Iquitos to buy them. This highlights both economic and geographical barriers to health services. Additionally, previous studies have shown that a lack of access to essential medicines can increase the use of and reliance on traditional/natural medical practices [25]. And indeed, some respondents of the current study indicated that they turned to home remedies to cure common illnesses if the health facility did not cure their child’s illness. Some respondents cited that they prefer to drink traditional/natural supplements over prenatal vitamins (4%), such as drinking masato (fermented cassava drink) and chuchuhuasi (sugar cane alcohol with plant root infusion). The research team questions the extent of alcohol that is consumed in this tradition, and further research is needed to explore the effects this practice may have.

The current study also evidenced a gap in the demand for contraceptive services. In the current study, most participants were aware they could obtain contraceptives for free at the health facility (90%). However, many had never spoken with someone at the health facility regarding family planning (30%), and many of the mothers have never sought contraceptives (28%). Another study in the region found that the main method for preventing unwanted pregnancy was periodic abstinence (41%) and of the pregnant women surveyed, 71% reported that their previous pregnancy was unwanted [48]. The two studies suggest that further outreach work is needed to improve the use of and demand for contraceptives in the region.

With regards to the adolescent mothers surveyed, few had heard of any adolescent health services (10%) or family planning services (10%). This is particularly concerning, as it indicates that the target population of these services is not aware that they are available and suggests that further outreach work is needed. Additionally, one respondent explained that some health facilities require parental approval for adolescents to obtain contraception, which can present a barrier to accessing family planning services. The research team investigated the barrier further by speaking with the local health facilities; some confirmed this as a requirement while others did not hold the same policy. This inconsistency is concerning in that it represents an important potential barrier for adolescents that want to prevent pregnancy but are unable to access family planning services. Finally, other research notes the strong influences of gender-power relations regarding the sexual and reproductive health decisions of adolescents, particularly in the Amazon context [49]. Further research is needed to better illustrate these important influences in the communities surveyed.

Lastly, it is important to note a potential under-utilization of services, requiring further study. Responses regarding health-seeking behavior for common childhood illnesses (diarrhea and respiratory illness) show that the health facility is the first line of treatment for nearly all mothers in the study. However, although the study shows that caregivers utilize the health facility when they determine treatment is needed, we do not know how frequently care is sought. If the caregivers have poor recognition sensitivity of common illnesses or underestimate the severity of the illnesses, there may be under-utilization. A previous study in Peru showed that the poorest stratum of the population attend the health facilities for childhood illnesses less than the more wealthy stratum [19]. Another study showed that sensitivity of recognition of illnesses like diarrhea, malaria, and pneumonia was low in impoverished populations [3]. Further study is needed in the local context to determine how frequently caregivers seek treatment from the health facility and at what point in the course of the illness they recognize it and deem it appropriate to seek that care.

### Implications for health policy

Policy makers can use the information to address the individual- and contextual-level factors affecting the health seeking behaviors of mothers with young children, tackling barriers and increasing utilization and effectiveness of services. The communities could benefit from campaigns to increase the understanding of vitamin supplements, vaccines, family planning, and the importance of prenatal checkups. Capacity building for health service personnel to ensure quality and welcoming patient interactions could also improve health service utilization. The local health facilities could be improved by increased staffing to decrease wait times, better supervision to ensure they are open during operating hours, and maintain stock of essential supplies and medicine in the health facilities. Finally, health authorities should implement policies that guarantee adolescents have access to family planning services throughout the region.

### Limitations and strengths

As with the majority of studies, the design of the current study is subject to limitations. The study was limited by having an incomplete representation of the types of communities in the region. The Amazon region houses a large portion of communities that are more distant from health facilities than those represented in the study. The communities included in the study may therefore represent a selection bias, due to their relative proximity to Iquitos. This relative proximity may also impact the reported health service utilization. Additionally, the participants included in the study were pointed out by the community health workers, and therefore already have some level of contact with the formal health sector. This may help explain the relatively high level of reported health service utilization. There may also be bias in responses due to social desirability bias; the participants may have told the surveyors what the participants believed the surveyors wanted to hear. Finally, the small sample size makes it difficult to generalize the quantitative data.

In terms of strengths, the study benefitted from a mixed-methods design, quantitatively capturing utilization of essential health services while also providing an opportunity for participants to describe individual- and contextual-level factors that might affect health seeking-behavior. The study also included a relatively high number of participants for a qualitative study, which helps ensure that the responses are representative of that community.

## Conclusion

Many of the findings in the current study reflect the insufficient conditions of health services. However, the study also found that many mothers have a positive outlook on the health services available to them, are proactive in the care for their child, and show knowledge of effective health practices. Most participants reported using the various health services available to them but flagged several issues, which affected overall satisfaction and accessibility.

In the study communities, most participants indicated that they utilize most of the maternal and child health services available to them in their community. They also reported overall satisfaction with the services. Nearly all mothers reported that they brought their child to child growth monitoring checkups, attended their prenatal health checkups, and seek care at the local health facility when their child is ill. However, among the issues mentioned, many participants reported that other mothers in the community do not utilize a variety of the health services due to embarrassment or fear, limited number of staff resulting in long wait times and inadequate operating hours in the health facility, impolite or rude providers, and low demand for family planning services. The study also found that many, especially adolescents, are unaware of the adolescent-specific service package available. The mixed-method study provides important insight into the use of local health services and the common perspectives that are hindering their further uptake at the community level in the Amazon region of Peru.

## Data Availability

The datasets generated and analyzed during the current study are available in the Figshare repository, 10.6084/m9.figshare.6837881

## Abbreviations

CHNRI: Child Health and Nutrition Research Initiative
DIRESA: Regional Health Directorate (“Direccion Regional de Salud” in Spanish)
PAHO: Pan American Health Organization
SIS: Comprehensive Health Insurance (“Seguro Integral de Salud” in Spanish)
UNICEF: United Nations Children’s Fund
WHO: World Health Organization
